# Failure of hospital employees to comply with smoke-free policy is associated with nicotine dependence and motives for smoking: a descriptive cross-sectional study at a teaching hospital in the United Kingdom

**DOI:** 10.1186/1471-2458-9-238

**Published:** 2009-07-15

**Authors:** Tom Parks, Clare VR Wilson, Kenrick Turner, Joel WE Chin

**Affiliations:** 1 University of Cambridge School of Clinical Medicine, Addenbrooke's Hospital, Hills Road, Cambridge, CB0 2SP, UK

## Abstract

**Background:**

Smoke-free policy aims to protect the health of the population by reducing exposure to environmental tobacco smoke (ETS), and World Health Organisation (WHO) guidance notes that these policies are only successful if there is full and proper enforcement. We aimed to investigate the problem of resistance to smoking restrictions and specifically compliance with smoke-free policy. We hypothesised that an explanation for non-compliance would lie in a measurable difference between the smoking behaviours of compliant and non-compliant smokers, specifically that non-compliance would be associated with nicotine dependence and different reasons for smoking.

**Methods:**

We conducted a questionnaire-based, descriptive, cross-sectional study of hospital employees. Seven hundred and four members of staff at Addenbrooke's Hospital, Cambridge, UK, completed the questionnaire, of whom 101 were smokers. Comparison between compliant and non-compliant smokers was made based on calculated scores for the Fagerström test and the Horn-Waingrow scale, and level of agreement with questions about attitudes. For ordinal data we used a linear-by-linear association test. For non-parametric independent variables we used the Mann-Whitney test and for associations between categorical variables we used the chi-squared test.

**Results:**

The demographic composition of respondents corresponded with the hospital's working population in gender, age, job profile and ethnicity. Sixty nine smokers reported they were compliant while 32 were non-compliant. Linear-by-linear association analysis of the compliant and non-compliant smokers' answers for the Fagerström test suggests association between compliance and nicotine dependence (*p *= 0.049). Mann-Whitney test analysis suggests there is a statistically significant difference between the reasons for smoking of the two groups: specifically that non-compliant smokers showed habitual smoking behaviour (*p *= 0.003). Overall, compliant and non-compliant smokers did not have significantly different attitudes towards the policy or their own health.

**Conclusion:**

We demonstrate that those who smoke in this setting in contravention to a smoke-free policy do so neither for pleasure (promotion of positive affect) nor to avoid feeling low (reduction of negative affect); instead it is a resistant habit, which has little or no influence on the smoker's mood, and is determined in part by chemical dependence.

## Background

Environmental tobacco smoke (ETS) is harmful to the health of those exposed[1-3]. Smoke-free policy aims to protect the health of the population by reducing exposure to ETS and World Health Organization (WHO) guidance notes that these policies are only successful if there is full and proper enforcement[4].

Others have established that there is poor compliance with the United Kingdom's National Health Service (NHS) smoke-free policy[5]. Small studies have evaluated the attitudes of NHS staff towards this policy finding limited support and poor compliance from employees[6], and a sense that the policy provided neither support nor motivation to those who wished to stop smoking[7].

We identified two groups amongst hospital staff who smoke: 1) those who are compliant with smoke-free policy and only ever smoke off the site, and 2) those who are non-compliant and continue to smoke on site. We focused on the latter group and aimed to ascertain why they appear to be resistant to policy. We hypothesised that this division might be attributable to a measurable difference in the smoking behaviour of each group. We postulated further that this factor would outweigh any apparent differences in the attitudes of the two groups towards the policy.

## Methods

### Setting

Addenbrooke's Hospital is a large NHS quaternary referral centre with 1,170 beds and 6,981 staff (2007/8), located in Cambridge, UK[8]. In accordance with the Government White Paper, *Choosing Health*[9], Addenbrooke's Hospital adopted a smoke-free site policy in January 2006 prohibiting smoking on site by staff, visitors and patients[10]. In addition, the *Health Act 2006 (Part 1)*, which legislates for a Smoke-Free England from July 1st 2007, brings the NHS in England under statutory requirements to prevent their employees and the public from smoking in "wholly or substantially enclosed areas" of their premises[11]. At the time of the study, monitoring and evaluation of compliance with the smoke-free policy at Addenbrooke's Hospital was limited, as has been observed at other NHS institutions[5]. Six months after data collection was complete, Addenbrooke's Hospital formally relaxed its smoke-free policy and reintroduced smoking shelters.

### Procedure

In March 2008 we performed a questionnaire-based, descriptive, cross-sectional study of Addenbrooke's staff. All hospital staff (n = 6,981) were eligible to complete a questionnaire over a 30-day period. Staff were made aware of the study through the hospital's Communications Department and a prize draw was offered as an incentive. The questionnaire could be completed either online, via the hospital intranet using Apollo[12] (an original, secure, online survey application) or as a paper copy, available to those members of staff who had no access to computers in order to maximise returns.

Respondents gave their informed consent to participate in the study and all data were anonymised at the point of collection.

The study was conducted as an audit and the Local Research Ethics Committee deemed a full ethical review to be unnecessary.

### Questionnaire Design

The key components of our questionnaire were recognised assessment instruments for smoking research: the Fagerström test[13] and the Horn-Waingrow scale[14].

Nicotine dependence was measured using the well-validated Fagerström test, a psychometric assessment tool consisting of six questions with answers that can be summed up for categorising nicotine dependence into minimally (score 0–3), moderately (score 4–6) and heavily (score 7–10) dependent groups[13,15-17].

We investigated reasons for smoking using the Horn-Waingrow scale, which is a 23-item Likert-style question set that has been validated in several studies[18-21]. We aimed to identify differences in smoking habit and motive between compliant and non-compliant smokers, which are important factors in addition to nicotine dependence when evaluating addictive behaviours[22]. There are six behaviours, none of which are mutually exclusive, and a respondent is scored between 2 and 30 depending on their level of agreement with each set of questions.

We also included pertinent questions from previous studies examining attitudes towards smoking policy[7,6]. Those respondents who reported smoking were divided into *compliant* smokers and *non-compliant* smokers on the basis of their    response to the question "When I am working at Addenbrooke's Hospital, I smoke on the site: More than twice a day/Once or twice a day/Once a week/Once a month/Never." Those who choose the answer "Never" were considered to be *compliant*.

A pilot study involving a preliminary test questionnaire design and wording was evaluated at a neighbouring district general hospital during July 2007, and received 53 responses; however, data were discarded from this pilot study, as the small sample size precluded any significant statistical conclusions. It emerged from this pilot study that a significant minority of hospital staff did not have computer access, and therefore a paper questionnaire was incorporated into the main study. We also modified the wording of a number of questions.

To avoid response bias, we distributed an identical set of questions on paper to certain groups of staff who have limited access to computer facilities at work. To encourage truthfulness and to avoid recall bias anonymity and dissociation from the Trust were emphasised. We considered that attitude towards the policy, health beliefs and demographic factors might modify or confound associations. To limit this, we included questions that would identify these factors. Questionnaires were excluded if questions concerning compliance, dependence and addiction were not complete.

### Statistical Considerations

Based on results from the pilot study, sample sizes for compliant and non-compliant groups were estimated as follows: a sample size of 30 individuals in each group has a power of 80% to detect a difference between means of 1.72 (on a scale of 0–10) a significance level of 0.05 (two-tailed), based on the Fagerström test. Our pilot study showed that smokers constituted 38% of total respondents (n = 53). Hence, we aimed to recruit at least 200 respondents. Any member of staff at the hospital could take part in the study and it was hoped that we would obtain a sufficient sample through voluntary participation.

The demographic information gathered from respondents was analysed and described for gender, age, job and ethnicity. Comparison between compliant and non-compliant smokers was made based on calculated scores for the Fagerström test, Horn-Waingrow scale and level of agreement with questions about attitudes. For ordinal data, a linear-by-linear association test was used to assess whether there was a significant difference between the two groups of smokers. For the Horn-Waingrow scale, the Mann-Whitney test was used to determine any significant differences in two non-parametric independent variables. For questions relating to attitudes, the Fisher's Exact test was used to test for any association between smoking status, compliance and agreement to the questions. The 95% confidence intervals (CI) for proportions were estimated by approximation to the binomial distribution and the use of exact methods. A *p *value of less than 0.05 was considered to be significant.

## Results

### Participants

Seven hundred and four members of staff completed and returned the questionnaire, three and a half times more than our minimum requirement. An additional 126 members of staff started but did not complete the questionnaire. The demographic composition of our sample was largely representative of the hospital's working population for gender, age, job profile and ethnicity. There were however differences: those aged 25 years or under were over-represented compared to those aged 26 to 45 years, men were over-represented and healthcare staff (professional and auxiliary) were under-represented (Table 1). Respondents represented one tenth of the total number of staff at the hospital.

**Table 1:** Socio-demographic characteristics of respondents and the population

		**Respondents**				**Population**
			
		*non-smokers*	smokers* frequency (%)		*total*	
					
**Socio-demographic characteristic**		frequency (%)	*compliant*	*non-compliant*	frequency (%)	frequency (%)
age	under 25	113 (18.9)	16 (23.5)	8 (25.8)	137 (19.6)	419 (6)
	
	26 – 45	300 (50.1)	41 (60.3)	16 (51.6)	357 (51.1)	4531 (64.9)
	
	over 46	186 (31.1)	11 (16.2)	7 (22.6)	204 (29.2)	2031 (29.1)

sex	male	169 (28.6)	15 (21.7)	8 (26.7)	192 (27.9)	1389 (19.9)
	
	female	421 (71.4)	54 (78.3)	22 (73.3)	497 (72.1)	5592 (80.1)

job title	healthcare professionals†	249 (41.6)	29 (42.0)	11 (34.4)	289 (41.3)	3609 (51.7)
	
	auxillary healthcare‡	87 (14.5)	4 (5.8)	3 (9.4)	94 (13.4)	1250 (17.9)
	
	estates	31 (5.2)	5 (7.2)	1 (3.1)	37 (5.3)	293 (4.2)
	
	contract ancillary	71 (11.9)	5 (7.2)	12 (37.5)	88 (12.6)	551 (7.9)
	
	clerical and managerial§	161 (26.9)	26 (37.7)	5 (15.6)	192 (27.4)	1278 (18.3)

ethnicity	caucasian	526 (87.8)	66 (95.7)	29 (90.6)	621 (88.2)	5892 (84.4)
	
	other	77 (12.8)	3 (4.3)	3 (9.4)	83 (11.8)	754 (10.8)

		**Fisher's exact test for significance**				
		
**Socio-demographic characteristic**		*smokers vs non-smokers *probability	compliant vs non-compliant* probability		non-smokers vs non-compliant* probability	non-smokers vs compliant* probability

age		**0.024||**	0.636		0.458	**0.028||**

sex		0.332	0.612		0.5	0.258

job title		0.164	**0.002||**		**0.007||**	0.09

ethnicity		0.065	0.378		**0.047||**	0.786

*Those respondents who reported smoking were divided into *compliant* smokers and *non-compliant* smokers on the basis of their response to the question "When I am working at Addenbrooke's Hospital, I smoke on the site: More than twice a day/Once or twice a day/Once a week/Once a month/Never." Those who choose the answer "Never" were considered to be *compliant*.

†Including doctors, registered nurses and other registered practitioners

‡Including all unregistered clinical staff e.g. healthcare assistants

§Including all administrative staff and non-clinical managers

|| Significant

In terms of reported smoking profile, 14.3% (95% Confidence Interval, CI, 12.0 – 17.1%) were smokers, 21.7% (95% CI 18.8 – 24.9%) were ex-smokers and 63.9% (95% CI 60.3 – 67.3%) had never smoked. This is consistent with the 2006 national figures obtained from the General Household Survey (22% smokers, 24% ex-smokers and 54% non-smokers)[23]. Smoking status was independent of sex, job title and ethnicity but respondents aged 46 years or over were less likely to smoke than younger respondents (*p *= 0.024).

Amongst those who smoked (n = 101), 69 were compliant while 32 were non-compliant with the hospital's smoke-free policy. Gender, age and ethnicity were similar between compliant and non-compliant smokers. There were occupational differences in compliance with the policy (*p *= 0.002). Specifically, contract ancillary workers were less likely to comply compared with others (*p *< 0.001) while clerical and managerial staff were more likely to comply (*p *= 0.036). Interestingly, healthcare professionals were neither more nor less likely to comply (*p *= 0.517).

### Nicotine dependence (Fagerström Test for Nicotine Dependence)

There were 69 compliant smokers and 32 non-compliant smokers. The mean (standard deviation, SD) Fagerström score (on scale 0–10) for compliant smokers was 2.91, (1.97); and for non-compliant smokers was 4.03 (2.31). Of the compliant smokers, 39% (95% CI 28% – 50%) were at least moderately dependent, compared with 59% (95% CI 42% – 75%) of non-compliant smokers (Table 2). Linear-by-linear association analysis of answers to the Fagerström test by the two groups allows us to exclude our null hypothesis that non-compliance would not be associated with nicotine dependence (*p *= 0.049). If absolute values are used the difference remains statistically significant (*p *= 0.021).

**Table 2:** Results of the Fagerström test for compliant and non-compliant smokers

	Nicotine dependence (Fagerström Score)*						
	
	*minimally dependent*		*moderately dependent*		*heavily dependent*		*total*
	frequency	percentage (95% CI)	frequency	percentage (95% CI)	frequency	percentage (95% CI)	frequency

*compliant*†	42	61 (49 – 72)	23	33 (23 – 45)	4	6 (2.3 – 14)	69

*non-compliant*†	13	41 (25 – 58)	15	47 (31 – 64)	4	12 (5 – 28)	32

*Chi-square (linear-by-linear association) = 3.860, ***p *= 0.049**

†Those respondents who reported smoking were divided into *compliant* smokers and *non-compliant* smokers on the basis of their response to the question "When I am working at Addenbrooke's Hospital, I smoke on the  site: More than twice a day/Once or twice a day/Once a week/Once a month/Never." Those who choose the answer "Never" were considered to be *compliant*.

### Reasons for smoking (Horn-Waingrow Scale)

There was a statistically significant difference between compliant and non-compliant smokers relating to habit (*p *= 0.003) using the Mann-Whitney test for non-parametric independent variables (Table 3). This allows us to exclude our null hypothesis that non-compliance would not be associated with any of the reasons for smoking. Habit is one of six reasons for smoking revealed by the Horn-Waingrow scale, none of the others – stimulation, pleasure, sensorimotor manipulation, psychological addiction and negative affect reduction – showed statistically significant differences. Non-compliant smokers scored higher for five out of the six reasons.

**Table 3:** Comparison of scores for Horn-Waingrow scale between compliant and non-compliant smokers

		**Reasons for Smoking (Horn-Waingrow Scale)**					
		
		*pleasure*	*stimulation*	SMM*	*habit*	*addiction*	*RNA*†
*compliant*‡	mean (SD§)	7.41 (2.10)	8.03 (2.57)	6.71 (2.61)	7.3 (2.00)	14.13 (4.25)	19.9 (4.78)

*non-compliant*‡	mean (SD§)	6.94 (2.51)	9.06 (3.46)	8.03 (3.18)	9.78 (3.99)	15.72 (4.99)	20.1 (6.11)
probability**||**		0.44	0.163	0.068	**0.003**¶	0.24	0.99

*Sensory Motor Manipulation

†Reduction of Negative Affect

‡Those respondents who reported smoking were divided into *compliant* smokers and *non-compliant* smokers on the basis of their response to the question "When I am working at Addenbrooke's Hospital, I smoke on the site: More than twice a day/Once or twice a day/Once a week/Once a month/Never." Those who choose the answer "Never" were considered to be *compliant*.

§Standard Deviation

**||**Mann-Whitney test

¶Significant

### Attitudes towards smoke-free policy and personal health

Based on a set of questions regarding attitudes toward the policy and personal health beliefs (Table 4), smokers displayed different attitudes compared with non-smokers. Smokers disagreed with the policy (*p *< 0.001), were less likely to agree that it protects people from passive smoke (*p *< 0.001) and felt they did not receive sufficient help from the hospital to quit smoking (*p *< 0.001). Predictably, those few respondents who did not care about their health were more likely to be smokers (*p *= 0.008).

**Table 4:** Agreement with the tobacco control policies

	**Respondents in agreement**			
	
	*non-smokers*	smokers*	
			
**Attitude question**	frequency (%)	*compliant *frequency (%)	*non-compliant *frequency(%)	
I am aware of this policy	601 (100.0)	69 (100.0)	32 (100.0)	

The hospital is right to have such a policy	510 (85.3)	25 (36.8)	11 (34.4)	

The policy protects people against passive smoke	313 (61.6)	24 (35.8)	15 (48.4)	

The policy is adequately enforced	124 (20.7)	13 (18.8)	15 (46.9)	

Smokers don't get enough help from the hospital if they want to quit	96 (16.1)	30 (43.5)	12 (37.5)	

Smoking damages my health	525 (87.1)	60 (87.0)	27 (84.4)	

I care about my health	593 (98.7)	66 (95.7)	28 (90.3)	

	**Fisher's exact test for significance**			
	
**Attitude question**	*smokers vs non-smokers *probability	compliant vs non-compliant* probability	non-smokers vs non-compliant* probability	non-smokers vs compliant* probability

I am aware of this policy	N/A†	N/A†	N/A†	N/A†

The hospital is right to have such a policy	**<0.001**‡	0.499	**<0.001**‡	**<0.001**‡

The policy protects people against passive smoke	**<0.001**‡	0.272	0.102	**<0.001**‡

The policy is adequately enforced	0.075	**0.004**‡	**0.001**‡	0.428

Smokers don't get enough help from the hospital if they want to quit	**<0.001**‡	0.365	**0.004**‡	**<0.001**‡

Smoking damages my health	0.451	0.473	0.409	0.55

I care about my health	**0.008**‡	0.27	**0.014**‡	0.094

*Those respondents who reported smoking were divided into *compliant* smokers and *non-compliant* smokers on the basis of their response to the question "When I am working at Addenbrooke's Hospital, I smoke on the site: More than twice a day/Once or twice a day/Once a week/Once a month/Never." Those who choose the answer "Never" were considered to be *compliant*.

†All participants agreed with "I am aware of this policy"

‡Significant

When comparing compliant and non-compliant smokers, differences were less obvious. Both groups were aware of the policy and disagreed with it as well as feeling that smokers did not get enough help from the hospital to quit their habit. Non-compliant smokers were more likely to agree that the policy was adequately enforced (*p *= 0.004) while compliant smokers echoed non-smokers in disagreeing with this statement (*p *= 0.428). In the main, both groups of smokers cared about their health and were aware that smoking was detrimental to it.

## Discussion

We present the first report – that we are aware of – in which established, questionnaire-based tools that describe a smoker's behaviour are applied to the problem of non-compliance with smoke-free policy. We hypothesised that the division of compliant and non-compliant smokers might be attributable to a measurable difference in the smoking behaviour of each group. Further we postulated that this factor would outweigh any apparent differences in the attitudes of the two groups towards the policy. We exclude our null hypothesis with the result that neither nicotine dependence nor reasons for smoking were independent of compliance. Although non-compliance was associated with agreement with certain attitude statements, overall it appears likely that the difference in the smoking behaviour would outweigh any apparent differences in the attitudes of the two groups towards the policy.

The study is limited by the size of our sample, which represents only one tenth of the eligible population. Larger responses would have been difficult to achieve in this setting, as effective communication within a sizeable teaching hospital can be difficult. It is, however, far larger than any previous studies of smoking on NHS hospital sites[7,6] and suitably powered. We speculate that response bias is inevitable as some individuals are more likely to complete a questionnaire than others. For example healthcare staff (professional and ancillary) may have prioritised (or been expected to prioritise) their time to clinical duties rather than completing the questionnaire and younger members of staff may have found it easier to access the online questionnaire. The over- and under-representation of particular groups is probably attributable to a complex combination of practical, psychological and social factors, particularly as smokers are a cohort who feel an increasing level of discrimination[24]. Despite anonymity and dissociation from their employer, recall bias will inevitably have affected the way the staff answered questions about compliance and smoking behaviour for fear of repercussions. We are further limited by our failure to include incomplete questionnaires in the analysis but, given there were only 35 smokers amongst the incomplete questionnaires and no method for handling missing data is without limitation[25], the impact of this is likely to be minimal.

With these limitations and the possibility of unidentified confounding factors, the implications of our explanation for non-compliance with smoke-free policy, based exclusively on these differences in nicotine dependence and habitual smoking, are rendered conjectural. Further, we did not examine any subgroups such as those who smoke on site only occasionally. Habitual smoking is a form of psychological addiction, which may coexist with chemical dependence, where there is no true reason for smoking except that it has become learned and automatic, with no influence on the smoker's mood or affect[14]. For example, the smoker may not even be aware of smoking and may even light one cigarette while another is still burning in an ashtray. A smoker's score in the Fagerström test correlates with the salivary cotinine levels and is a marker of chemical dependence[26-28]. We believe that our dataset is sufficiently robust to demonstrate that those who smoke in this setting in contravention to a smoke-free policy do so neither for pleasure (promotion of positive affect) nor to avoid feeling low (reduction of negative affect); instead it is a resistant habit, which has little or no influence on mood and is determined in part by chemical dependence.

This explanation for non-compliance with smoke-free policy is relevant to individual NHS hospitals developing new approaches towards tobacco control. We would argue that these findings apply to all such hospitals because all hospitals have a similar population of employees and all NHS hospitals introduced analogous smoke-free policies following guidance from the UK's Health Development Agency[29]. The role of health services in implementing smoking policies has long been cited[30] but it remains controversial. From 1992 the Joint Commission on Accreditation of Healthcare Organizations in the USA has required all hospital buildings (but not their grounds) at accredited institutions to be smoke-free[31]. The concept of a totally smoke-free hospital (buildings and grounds) can be traced back to the late 1980s and early 1990s and Johns Hopkins Hospital in Baltimore[32]. More recently the European Network of Smoke Free Hospital and Health Services (ENSH) has also promoted completely smoke-free hospital grounds[33]. In Spain, this has lead to the successful implementation of smoke-free hospital sites[34] and with the introduction of nationwide smoke-free legislation ETS exposure at these institutions is decreasing[35]. Despite these successes, there remain difficulties. For example, in the USA, concerns pertaining to employee retention and public relations, although not borne out in the research, have been highlighted[36].

In 2004, the UK Government's Public Health White Paper, *Choosing Health*[9], set out the Government's intention for the NHS to become a model employer in supporting and promoting the health of its staff. This included the requirement that NHS hospitals become smoke-free by the end of 2006 but poor compliance continues to be observed across the country[5]. It follows that there is a major incentive to address public health issues amongst the 1.3 million NHS employees (2% of the UK's population) who are from all socio-economic classes and ethnic groups. Importantly, it may be possible to generalise successful strategies to the entire UK population and beyond.

On the basis of this we would advocate further observational studies to examine the impact of proactive interventions that specifically address nicotine dependence and psychological addiction amongst non-compliant smokers. For example, there may be merit in screening the working population for individuals with particular smoking behaviours and offering evidence-based workplace interventions for smoking cessation[37]. This might not only improve their compliance but also, more importantly, increase the likelihood that they quit smoking.

## Conclusion

We have shown that measurable differences in smoking behaviour provide a potential explanation for non-compliance with smoke-free policy. Although the primary aim of smoke-free policy is to protect the health of the entire population by reducing exposure to ETS, an arguably more important effect is a reduction in smoking prevalence and the consumption of tobacco which protects the health of the individual smoker[38]. However, even in California, which, since the 1990s, has led the way in terms of smoking initiation and cessation rates, 15.2% of the population continued to smoke in 2005[39]. It appears that a proportion of the population is resistant to even the most comprehensive tobacco control programme. It is crucial, therefore, that future research asks why that proportion of individuals continue to smoke despite the increasing restrictions that are in place.

## Competing interests

The authors have no competing interests to declare. None of the authors smoke but all work, at times, on the Addenbrooke's Hospital site.

## Authors' contributions

All four authors contributed equally to the writing and editing of the manuscript and all authors read and approved the final manuscript. TP and CW suggested investigating the problem of smoke-free policy at Addenbrooke's Hospital and designed the study in conjunction with KT and JC. TP drafted the original proposal, gained the necessary permissions and carried overall responsibility for all aspects of the study. CW facilitated production and data entry of the paper questionnaire and was responsible for negotiating with the various departments at Addenbrooke's Hospital. KT wrote the database software (APOLLO) and website and (in conjunction with TP) was responsible for promoting the study at the hospital site. JC performed the statistical calculations on SPSS and developed the statistical methods with advisers at the Centre for Applied Medical Statistics, University Department of Public Health and Primary Care.

## Author information

All four authors are final year medical students at the University of Cambridge. This work was the initiative of these four students and neither a compulsory component of their course nor supervised by any one individual. Instead the four students sought advice from a number of senior members of the University of Cambridge, Cambridge University Hospitals NHS Foundation Trust, the British Thoracic Society and Cancer Research UK.

## Pre-publication history

The pre-publication history for this paper can be accessed here:


